# Author name disambiguation based on heterogeneous graph neural network

**DOI:** 10.1371/journal.pone.0310992

**Published:** 2025-02-26

**Authors:** Ge Wang, Zikai Sun, Weiyang HU, MengHuan Cai

**Affiliations:** College of Intelligent Equipment, Shandong University of Science and Technology, Taian, Shandong, China; Indiana University Bloomington, UNITED STATES OF AMERICA

## Abstract

With the dramatic increase in the number of published papers and the continuous progress of deep learning technology, the research on name disambiguation is at a historic peak, the number of paper authors is increasing every year, and the situation of authors with the same name is intensifying, therefore, it is a great challenge to accurately assign the newly published papers to their respective authors. The current mainstream methods for author disambiguation are mainly divided into two methods: feature-based clustering and connection-based clustering, but none of the current mainstream methods can efficiently deal with the author name disambiguation problem, For this reason, this paper proposes the author name ablation method based on the relational graph heterogeneous attention neural network, first extract the semantic and relational information of the paper, use the constructed graph convolutional embedding module to train the splicing to get a better feature representation, and input the constructed network to get the vector representation. As the existing graph heterogeneous neural network can not learn different types of nodes and edge interaction, add multiple attention, design ablation experiments to verify its impact on the network. Finally improve the traditional hierarchical clustering method, combined with the graph relationship and topology, using training vectors instead of distance calculation, can automatically determine the optimal k-value, improve the accuracy and efficiency of clustering. The experimental results show that the average F1 value of this paper’s method on the Aminer dataset is 0.834, which is higher than other mainstream methods.

## Introduction

With the dramatic increase in the number of published papers and the continuous advancement of deep learning techniques, research on name disambiguation is at an all-time high, the number of authors of papers is increasing every year, and the situation of authors with the same name is intensifying, therefore, it is a great challenge to accurately assign the newly published papers to their respective authors. In real life, the phenomenon of same name is common. And when a digital library user searches for a particular author’s name, he or she may see a mixture of results by different authors with the same author’s name between these results, and distinguishing between the two is an important prerequisite for improving the quality of digital library services and content quality. The general task of author disambiguation is to associate publications with the same name or highly similarly spelled names to different human entities. However, current methods suffer from the following shortcomings: (1) Existing methods suffer from insufficient feature utilisation. Typically, these methods only single-handedly utilise paper text or relational attributes, failing to effectively mine and fuse multiple types of features that are potentially valuable for the author name disambiguation task. (2) Using only local information. Existing methods, when using the network embedding-based approach for author name disambiguation, tend to construct homogeneous networks only for the local paper data corresponding to the name that generates the ambiguity, without effectively utilizing the rich semantic information in the global heterogeneous information network, which leads to inefficient use of global information and limits the performance of the disambiguation model. In response to the above mentioned deficiencies, in this paper, we improve the above deficiencies of the existing author name disambiguation methods, design the extraction of fusion of multiple types of features, the use of our graph convolution module to train the information splicing, which is able to get a better representation of the features, the construction of a multi-attention-head heterogeneous graph convolutional attention network way to learn not to use the type of nodes and edges between the rich interaction relationship to address the characteristics of global heterogeneous information network, Finally we improved the traditional hierarchical clustering method by combining the relationships and topologies between graphs such as relational semantics with hierarchical clustering for disambiguation of homonymous authors. And the experimental comparison with several baseline methods was conducted, and the experimental results show that the average F1 value of this paper’s method with only three paper features on the Aminer dataset is 0.834, which is higher than that of other mainstream methods.

## Related work

Author name ambiguity is a common challenge in academic literature databases and digital libraries. Some authors in the real world have the same name (Aman, 2018; Kim, 2020; Shoaib et al., 2020) [1, 2]. This problem is very challenging in literature databases because, for example, as of March 2023, there are about 40K articles in Microsoft Academic Graph (MAG) that cite the work of “Wang Wei”, and the problem of acronym name ambiguity is even more significant. Although databases such as MAG and AMiner provide disambiguating author identifiers (IDs), the performance of author identifier systems created based on the author name disambiguation (And) method is far from satisfactory for databases of millions in size (Zhang et al., 2020) [3]. After years of research, the authors have introduced a variety of different methods within the field of name elimination. For example, a variety of classification models are used to learn the two-by-two similarity function, including Naive Bayes [4], Logistic Regression [5], Support Vector Machines, Decision Trees, Random Forests (RF) [6], Deep Neural Networks (DNNs) [7] and Gradient Augmented Trees (GBTs), etc. Qiao et al [8] use DNNs with hand-crafted features, whereas zhang et al [9] utilise DNNs to learn feature representations from bag-of-words vectors to learn feature representations from bag-of-words vectors. Bertrand M et al [10] first learn the representation of each record using DNN and then improve it by an autoencoder, where the encoder is constructed based on the similarity between records. Meanwhile, some studies want to use unsupervised methods [8, 11] to solve the problem of author name disambiguation, and algorithms such as DBSCAN [11] and hierarchical clustering [12] have been used for this purpose. For example, Liu et al. [13] and Kim et al. [14] used the similarity between a pair of records with the same name to disambiguate author names on the PubMed dataset. Zhang et al. [15] used Recurrent Neural Networks (RNN) to predict the number of authors who published only one paper in the Aminer dataset, and in this direction they proposed a two-stage approach applied to the DBLP dataset. stage approach, in which the first stage is to divide the author records into different clusters and then disambiguate each cluster separately. Wu et al. [13] used Shannon entropy to fuse features such as the affiliation and content of the papers to generate a matrix that represents the two-by-two correlation of the papers, while Hierarchical Agglomerative Clustering (HAC) was used to predict the number of authors who have published only one paper in the Aminer dataset. HAC) uses this matrix to disambiguate author names in the dataset. In addition, supervised methods [4, 16] have been widely used in research, mainly applied after clustering together blocks of authors sharing the same name. Han et al. [4] proposed two supervised learning methods to disambiguate authors in the literature. For example, in the case of a selected reference, the first method uses a plain Bayesian model to find the author block with the maximum posterior probability. The second approach uses a support vector machine approach to classify references from DBLP to the original author. Sun et al. [17] used heuristic features such as the percentage of citations collected by the author’s name variable and made this to remove ambiguities in common author names. Additionally, neural networks were used to verify that two citations were close enough to determine if the two citations were published by the same target author. In [18], they propose an entity resolution system called “Deeper Levels”. This system uses a combination of Bidirectional Recurrent Neural Networks (BRNN) and Long Short-Term Memory (LSTM) as hidden units to generate distributed representations of each tuple in order to capture the connections between them. In summary, although there are some feature-based and connection-based author name disambiguation methods have been proposed but they tend to have problems such as insufficient use of the features and using only local information, this paper addresses the shortcomings of the existing author name disambiguation methods, and designs an author name disambiguation method that can fuse multiple types of features and use the construction of a heterogeneous graph convolutional network to solve the characteristics of the global heterogeneous information network. Specifically, our work as follows:

1. Proposing an author name disambiguation method based on the fusion embedding of semantic and relational features, we fuse and embed the extracted semantics and relations, which further condenses the aggregated information, transforms the high-dimensional sparse data in the graphs into the low-dimensional sparse data, and preserves the topology between individual graphs.
2. A global heterogeneous graph convolutional attention neural network (RA-HNet) is proposed, which uses a meta-path correlation weight sampling strategy in the network to fully fuse local and global information, and improves the structure of the graph neural network by adding a graph attention mechanism to the network in order to improve the network’s ability to learn the expression of the features, and lightens the network so that the network is able to learn the global variables while speeding up the training speed of the network, we also experimented on the sensitivity of the newly added multi-head attention parameter, and finally used the improved hierarchical clustering (RHAC) for clustering, our clustering algorithm can not specify the number of clusters, and through the iterative updating of the algorithm to determine the optimal k value automatically, and at the same time, can retain the topology of the graph, the experiments show that our method has a certain degree of superiority.
3. In this paper, a large number of comparative and ablation experiments have been conducted to demonstrate the superiority of this paper’s method in terms of modeling effect, clustering algorithm prediction accuracy, and clustering algorithm efficiency.

## Methods

In our work, thesis data is regarded as node information. A network linking the node information is constructed, and the network is used to save and express the relationship information between databases. Due to the diversity of the attributes of the relational information of thesis data, the general isomorphic network cannot save the information at the same time. We solve this problem by constructing a heterogeneous graph convolutional network.

### Definition 1 (Heterogeneous network)

A heterogeneous network refers to a graph *G* = (*V*, *L*, *N*, *R*) in which there is more than one class of nodes and more than one class of edges, and the sum of their classes is greater than 2. i.e., in a heterogeneous network, a heterogeneous graph is represented by a set of nodes *V* = (*v*_1_, *v*_2_……*v*_*n*_) and a set of edges *L* = (*l*_1_, *l*_2_……*l*_*n*_), where nodes *v* ∈ *V*, edges *l* ∈ *L*, and there are two mapping functions in a heterogeneous graph using Γ_*n*_ to denote the set of nodes, Γ_*i*_ to denote the set of edges, and each node corresponds to the node mapping function Φ_*n*_ : *L* → Γ_*n*_ and each edge corresponds to the edge mapping function Φ_*j*_ : *L* → Γ_*j*_, i.e., |*N*| + |*L*| ≥ 2.

### Definition 2 (Relational heterogeneous network)

In a heterogeneous network, each relation within the network has a weight, and for the relation *m* ∈ *M*, its value can be represented by |*m*|. In the Relational Heterogeneous Graph Convolutional Network (RA-HGCN) constructed in this paper, One type of node and three types of relations to construct this network are used, In the later part, we use RA-HGCN to represent our relational heterogeneous network. We take the published papers as the nodes of the heterogeneous network, and the three kinds of relations include Co-Reliable Author, Co-pub place and ReTitle, which will be explained in the following: Co-reliable Author Each published paper will have one Each published paper will have one or several authors, so when we want to disambiguate the author’s name is n, the author may be related to more than one paper, in order to eliminate the ambiguity of the author’s n, assume that two of the published papers $P_{1}^{n}$ are $P_{2}^{n}$ and, and the set of authors of these published papers are expressed as *N*_1_ and *N*_2_ respectively, so that $N_{1}^{\prime }$ means that *N*_1_ does not include the set of authors n; if the intersection set of and is null $N_{1}^{\prime }\cap N_{2}^{\prime }\neq ⌀$, then it means that in addition to the N, there are other co-authors between the two published papers, co-author weight is the weight of the co-authors. If the intersection of $N_{1}^{\prime }$ and $N_{2}^{\prime }$ is empty then it means that there are other co-authors between these two published papers besides n. The weight of the co-authors is $Q=N_{1}^{\prime }\neq N_{2}^{\prime }$. In this paper, we set a threshold value for the weights, and those exceeding this threshold value are defined as reliable co-authors.

#### Co-pub place

If two papers are published in the same conference or journal, it creates a co-pub place relationship between the two papers, generally the same paper will only be published in one journal or conference, so if there is a co-pub place relationship between the two papers then we define their value as 1.

#### ReTitle

A paper usually contains many keywords to summarise the main content of the paper, then for these keywords c, we first remove the stop words between, and then each keyword is converted to its stem, which forms a key phrase *T*_*p*_, then for two papers *A*_1_ and *A*_2_, if their key phrases have an intersection |*T*_*p*_1 ∩ *T*_*p*_2| ≠ ⌀, there is a Relevant Title relationship between them, with the weight of |*T*_*p*_1 ∩ *T*_*p*_2|.

**Fig 1:**
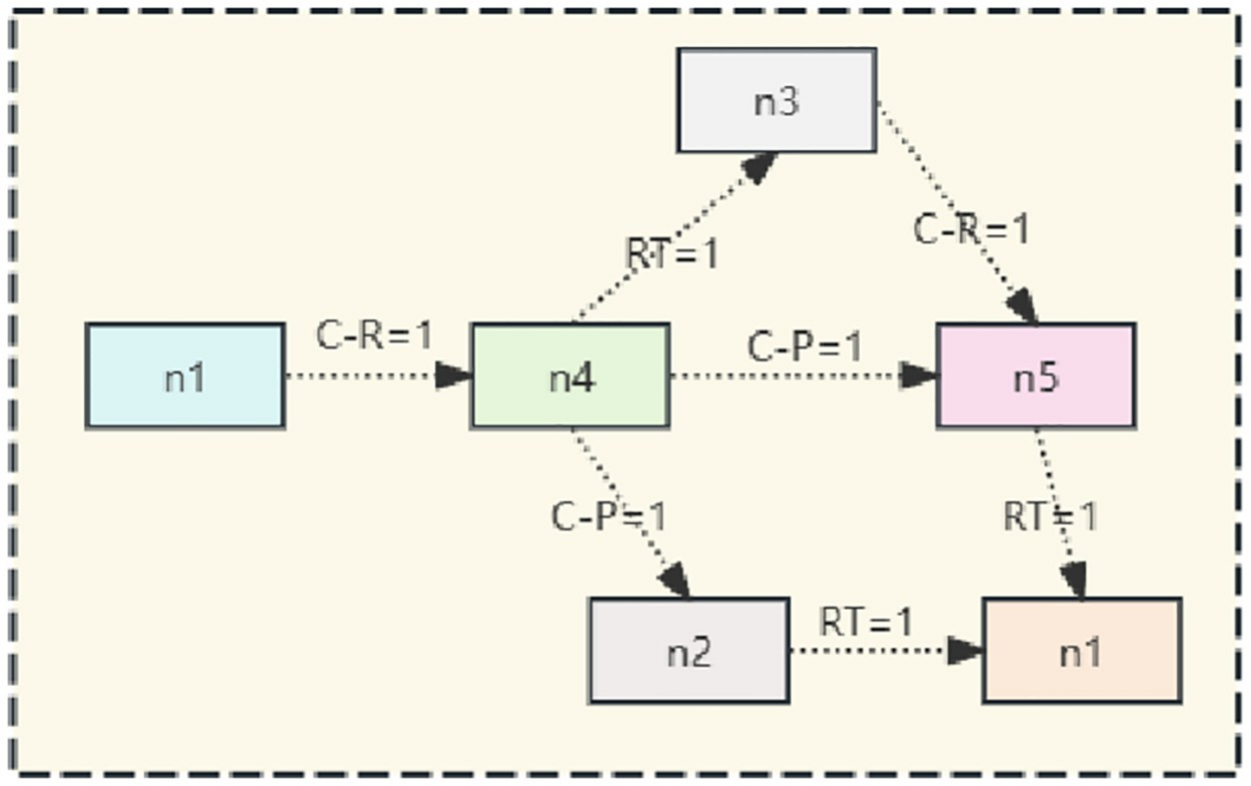
Random walk sampling methodology (in the figure C-R stands for co-reliable authors, C-P stands for co-publication sites and RT stands for relevant titles).

In our work, we use these three common attributes to construct a heterogeneous graph convolutional network, in which two nodes are connected by multiple undirected relations, through which we can find the connecting path between two nodes. For example, in Fig 1, there is a path $n_{1}\overset{c-r=1}{\to }n_{4}\overset{c-p=5}{\to }n_{5}$ between nodes *N*_1_ and *N*_5_, which can be interpreted as *N*_1_ and *N*_4_ between another paper by an author and *N*_5_ published in the same place.

### Definition 3 (Meta-path)

In our heterogeneous network G=(V,L,N,R), a meta-path can be denoted as $n_{1}\overset{r1}{\to }n_{2}\overset{r2}{\to }\overset{r3}{\to }\ldots \overset{rm}{\to }n_{m+1}$ where *n*_1_, *n*_2_, …*n*_*m*+1_ stands for the nodes and *r*_1_, *r*_2_, …*r*_*m*+1_ stands for the relationship between the nodes.

### Introduction of 3 PROPOSED MODEL

In this section, we present our heterogeneous graph convolutional network embedding method, which encodes the content and relationships of the paper separately and then clusters them using a graph-based clustering algorithm.

### Thesis network embedding section

In this section, we present the graph convolutional embedding module (RA-HNet), it gets the nodes by running on the neighborhood of a local graph, fuses semantics and relations between nodes using the fusion embedding module, we embed the fused features into the network layer, In the following part, we use RA-HNet to represent our graph volume product embedding module.

**Fig 2:**
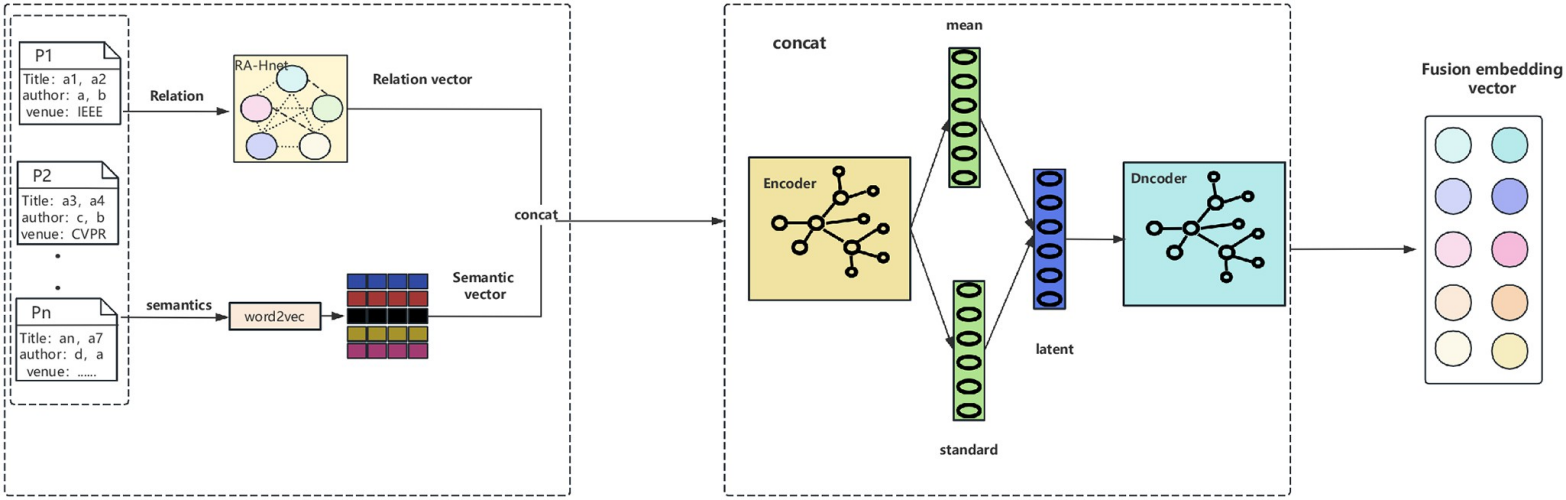
Fusion embedded modules.

Our fusion embedding module is shown below in Fig 2, this module includes an Encoder and a Decoder and an error function in between, the core idea of the encoder is to use the encoder to encode the data into mean and variance vectors, train the vectors to reconstruct the error so that the distribution in the latent space is close to the standard normal distribution, and use the decoder to decode it back to the original space. The output vector after training is the spliced feature vector. Given a graph G=(V,L,R), where V denotes the set of nodes, L denotes the set of edges, and R denotes the set of relations. Each node in the graph represents a published paper with keywords, authors and other information. Firstly node initialisation is performed, for the nodes of the published paper *p*_*i*_ ∈ *V*, we use Word2vec method to encode their information into a fixed length feature vector $u_{i}^{2}$, As shown in the following (1):

$$\begin{array}{c} u_{i}^{l}=\text{LeakyReLU}(a\sum_{r\in R}\sum_{j\in N_{j}^{r}}\frac{1}{c_{ij}^{r}}u_{j}^{l}w_{r}^{l}) \end{array}$$ (1)

In the formula $u_{i}^{l}\in R_{i}^{l\times m}$ is the hidden state of node *p*_*i*_ in the layer *t* of the relational network, and *m*^*t*^ is the dimension represented by this layer. In our experiments, we set *a* = 0.001 and LeakyReLU(⋅) = max(0, ⋅), $N_{i}^{r}$ to represent the set of neighbours cues of node *p*_*i*_ when the relation *r* ∈ *R* is in place, $c_{ij}^{r}=\sqrt{k_{i}^{r}k_{j}^{r}}$ represents the normalisation constant of the edge (*p*_*i*_, *p*_*j*_), while *ϕ*(*p*_*i*_, *p*_*j*_) = *r* represents the type of the edge is r, and $K_{i}^{r}$ represents the sum of the weights of the relations that are connected to *p*_*i*_ and have a relation type of $r\cdot W_{r}^{l}$. It is the relationship and layer specific trainable weight matrix that is used to ensure that the representation of a node can be mapped to the next layer. In order to ensure that each layer can have an effect on the nodes in the next layer, we assume that each node has a single connection to their neighbouring nodes with a weight of 1 to ensure that information can be passed between layers.

Then we define L convolutional layers in the RA-HGCN model, in each convolutional layer, the output of the previous layer is the input of the next layer, $u_{i}^{0}$ represents the initial features of the input nodes of the first layer, and for each thesis node *p*_*i*_, the RA-HGCN model embeds its textual information into $u_{i}^{0}$ and encodes it with its local neighbourhood on the graph G into *u*_*i*_, As shown in the following (2):

$$\begin{array}{c} u_{i}=u_{i}^{L}=\varphi \left(u_{i}^{0},G\right) \end{array}$$ (2)

*φ* represents the parameters of RA-HGCN, in each convolutional layer of RA-HGCN, each node receives the information of its one-hop neighbours to update the representation, and the neighbour information of each node under the same relation type shares the same transformation parameters. When multiple convolutional layers are stacked, the one-hop neighbour information received by each node already includes the neighbour information of the previous convolutional layer, and through the L-layer, a node can receive information from at most L-hop distances of its multi-relationship neighbours.

### Graph multiple attention mechanisms

Since the transformed information is sparse and not conducive to our distinction between authors with the same name, in order to enable our network to better aggregate information between neighboring nodes, combine the semantics and relationships between paper nodes, and improve the embedding ability of the nodes to efficiently transform high-dimensional data from published papers. In order to enable our network to better aggregate information between neighbouring nodes, combine the semantics and relationships between paper nodes, and improve the embedding ability of nodes to efficiently transform high-dimensional data in published papers. We adds the attention mechanism into the RA-HGCN model for information aggregation between publication nodes, as well as to learn the magnitude of the influence between each different neighbouring nodes. We firstly splice the first-order neighbor information of the current paper node and obtain the importance of the current neighbor node by transforming it as, As shown in the following Eq (3):

$$\begin{array}{c} \beta_{ij}=\sigma \left(a^{T}\cdot concat\left(v_{i},v_{j}\right)\right) \end{array}$$ (3)

Where, *β*_*ij*_ represents the degree of influence of the neighbour node *j* on the current node *i*, *v*_*i*_*v*_*i*_ ∈ *wD*. *σ* is the activation function, *a* is the weight vector, and concat denotes the splice operation, using the splice operation can join the first and last of the two vectors to form a new vector, and use Eq (4) to aggregate the weighted features between neighbours and update the node

$$\begin{array}{c} v_{i}^{\prime }=\sigma \left(\sum_{j\in \Omega }a_{ij}v_{j}\right) \end{array}$$ (4)

Formula *Omega* denotes the neighbouring nodes in the convolutional graph that are directly connected to the node *i*. In addition, since a single graph attention head does not aggregate node information enough and may not be able to take into account the influence relation of the whole network, for this reason we added multiple attention heads to the network in order to enhance the learning of node information in the graph, As shown in the following Eq (5).

$$\begin{array}{c} v_{i}^{\prime }=concat_{k=1}^{K}\left(\sigma \left(\sum_{j\in \Omega }a_{ij}^{k}\nu_{j}\right)\right) \end{array}$$ (5)

The k in the formula *a*^*k*^ represents the k attention head. In order to further condense the information of published papers, this paper designs a new fully-connected layer that uses the fully-connected layer to learn different weights as well as to classify co-reliable authors.

$$\begin{array}{c} y=W_{2}\left(W_{1}v_{i}^{\prime }+b_{1}\right)+b_{2} \end{array}$$ (6)

Where *W*_*i*_ represents the parameters of the fully connected layer and *b*_*i*_ is the bias term of the connected layer. In simple terms, if a path in the convolutional network has a positive effect on the disambiguation, then the parameter value of this path will become larger, and finally the generating vector *y* is calculated by Eq (6), and the *y* vector contains the characteristics of the nodes in this path as well as the path information, etc., and finally the cross-entropy loss function is used to get the optimal embedding of the literature nodes through cross-entropy, and the loss function is shown in Eq (7):

$$\begin{array}{c} loss=-\sum_{p_{i}^{a}p_{j}^{a}\in p^{a}}A_{ij}\cdot lod\left(y_{i}^{T}y_{j}\right) \end{array}$$ (7)

### Random sampling of association weights for meta-paths

The goal of this paper is to train a convolutional network (RA-HGCN) in such a way that the network is able to encode a high-quality representation of the nodes of each published paper. Inspired by the classical approaches of network embedding methods DeepWalk [19] and Metapath2Vec [20], which both employ a random wandering strategy and a skip-gram model, to learn and represent the nodes in the network. We propose an associative weight random sampling strategy for metapaths for sampling paths on weighted heterogeneous networks.

**Fig 3:**
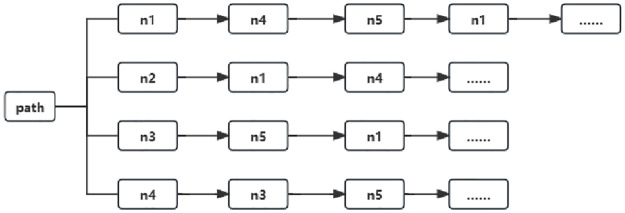
Example of random sampling.

Two nodes *n*_*i*_ and *n*_*j*_ can be connected by multiple undirected relations between them, and a sequence of connected nodes based on these relations can be seen as a path to the between *n*_*i*_ and *n*_*j*_. A path *n*_1_ → *n*_4_ → *n*_5_ exists between *n*_1_ and *n*_5_ as shown in Fig 3 as an example of partial random sampling in a network. The correlation of nodes in heterogeneous relationships can be captured using our method, while in sampling the paths we consider the relationship weights in the network. Intuitively it means that the higher the relationship value between two nodes, the higher the similarity; and in each step, as the executor moves towards its neighbours, the higher the relationship value between the current node and the neighbouring node, and the more likely it is to be sampled.

As an example, given G=(V,L,R), the path consists of $P=(p^{t}\overset{r_{1}}{\to }p^{t+1}\overset{r_{2}}{\to }\ldots \overset{r_{m}}{\to }p^{t+m+1})$, where *p*^*t*^ ∈ *V*, *r*^*i*^ ∈ *R*, represents the length of the relationship between *p*^*t*^, *p*^*t*+*m*+1^, the probability of transfer at step length t, As shown in the following Eq (8):

$$\begin{array}{c} p\left(p^{t+1}|p^{t},P\right)=\{\begin{array}{cc} \frac{\left|(p^{t},p^{t=1})\right|}{\left\|L^{t}\left(r_{i}\right)\right\|},\left(p^{t},p^{t=1}\right)\in L & \phi \left(p^{t},p^{t=1}\right)=r_{i}\\ 0,(p^{t},p^{t=1})\in L & \phi \left(p^{t},p^{t=1}\right)\neq r_{i}\left(p^{t},p^{t=1}\right)\notin L \end{array} \end{array}$$ (8)

where *r*_*i*_ ∈ *R*, is the next relation of *ϕ*(*p*^*t*−1^, *p*^*t*^), and if it is the first node, then *i* = 1, |*p*^*t*^, *p*^*t*+1^| denotes the weight of the relation between the two, and *L*^*t*^(*r*_*i*_) denotes the set of relations between *p*^*t*^, *r*_*i*_. Specifically, we first select a node in the RA-HGCN as the start node in the path and generate a meta-path of length l, and then use the last node in the network as the start node of another path. In this way, each random walk recursively samples nodes in the network and finally generates a long path of fixed length guided by p. Compared to the random walk strategy, this strategy is able to effectively avoid the influence of bias caused by the central node and the bias of the number of different relations. In addition to this the strategy in this paper also considers the possibility of diverse permutations between paths, as well as the multiple relationships between individual nodes in the path and the weights between individual relationships, which makes the strategy in this paper able to retain the heterogeneous relationships between nodes in the generated paths very well.

### A weighted heterogeneous jump graph model for semantics and relations

For the paths generated in the previous section, in this paper we use a weighted heterogeneous jump model (RAHG-skip gram) to learn published papers in the network. Given a G=(V,L,R), our goal is to efficiently learn node representations by maximising the probability that any node *p*_*i*_ has its neighbour *p*_*c*_, As shown in the following Eq (9):

$$\begin{array}{c} \text{arg}\;\underset{\theta }{\text{max}}\;\sum \sum_{p_{ier}}\sum_{r\in R}\sum_{p_{c}\in N_{r}\left(p_{i}\right)}\left(p_{c},p_{i}\right)|\;\text{log}\;p\left(p_{c}|p_{i},\theta \right) \end{array}$$ (9)

Where *N*_*r*_(*p*_*i*_) denotes the set of neighbours of *p*_*i*_, ∀*p*_*c*_ ∈ *N*_*r*_(*p*_*i*_), (*p*_*c*_, *p*_*i*_) ∈ *r*, *θ* denotes the RA-HGCN model of this paper, and the relation weight of the function |*p*_*c*_, *p*_*i*_| represents a weight which ensures that neighbours with correlation with have higher values as well as higher output probabilities, while our softmax function is defined As shown in the following Eq (10):

$$\begin{array}{c} p\left(p_{c}|p_{i},\theta \right)=\frac{\text{exp}\;(u_{i}\cdot u_{c})}{\sum_{j=1}^{D}\;\text{exp}\;\left(u_{i}\cdot u_{j}\right)} \end{array}$$ (10)

Where *u*_*i*_ represents the embedded network vector after the initial features of *p*_*i*_ are encoded by RA-HGCN in Eq (2), and then this paper adopts the negative sampling method which is popular in current research to improve the efficiency of optimisation, and the probability can be approximated and defined as in this paper’s treatment, As shown in the following Eq (11):

$$\begin{array}{c} \text{log}\;p\left(p_{c}|p_{i},\theta \right)\approx \;\text{log}\;\sigma \left(u_{i}\cdot u_{c}\right)+\sum_{p_{j}\in D_{neg}}\;\text{log}\;\sigma \left(-u_{i}\cdot u_{j}\right) \end{array}$$ (11)

where $\sigma \left(X\right)=\frac{1}{1+e^{-x}}$ represents the sigmoid function and $D_{neg}^{i}$ represents the set of negative samples in the path calculated by probability.

In the method of this paper, by selecting different initial nodes, a set of random path sets can be obtained, which is denoted by RW in this paper. And the frequency of their appearances is proportional to the frequency of their appearances in the context of the target node and the degree of relationship. Based on this situation, the maximisation of the objective can be approximated as equal to the minimisation of the loss function, As shown in the following Eq (12):

$$\begin{array}{c} L=-\sum_{w\in RW}\sum_{p_{i\in w}}\sum_{p_{c}\in C_{i}}\;\text{log}\;p\left(p_{c}|p_{i},\theta \right)+\lambda {\left\|\theta \right\|}^{2} \end{array}$$ (12)

**Fig 4:**
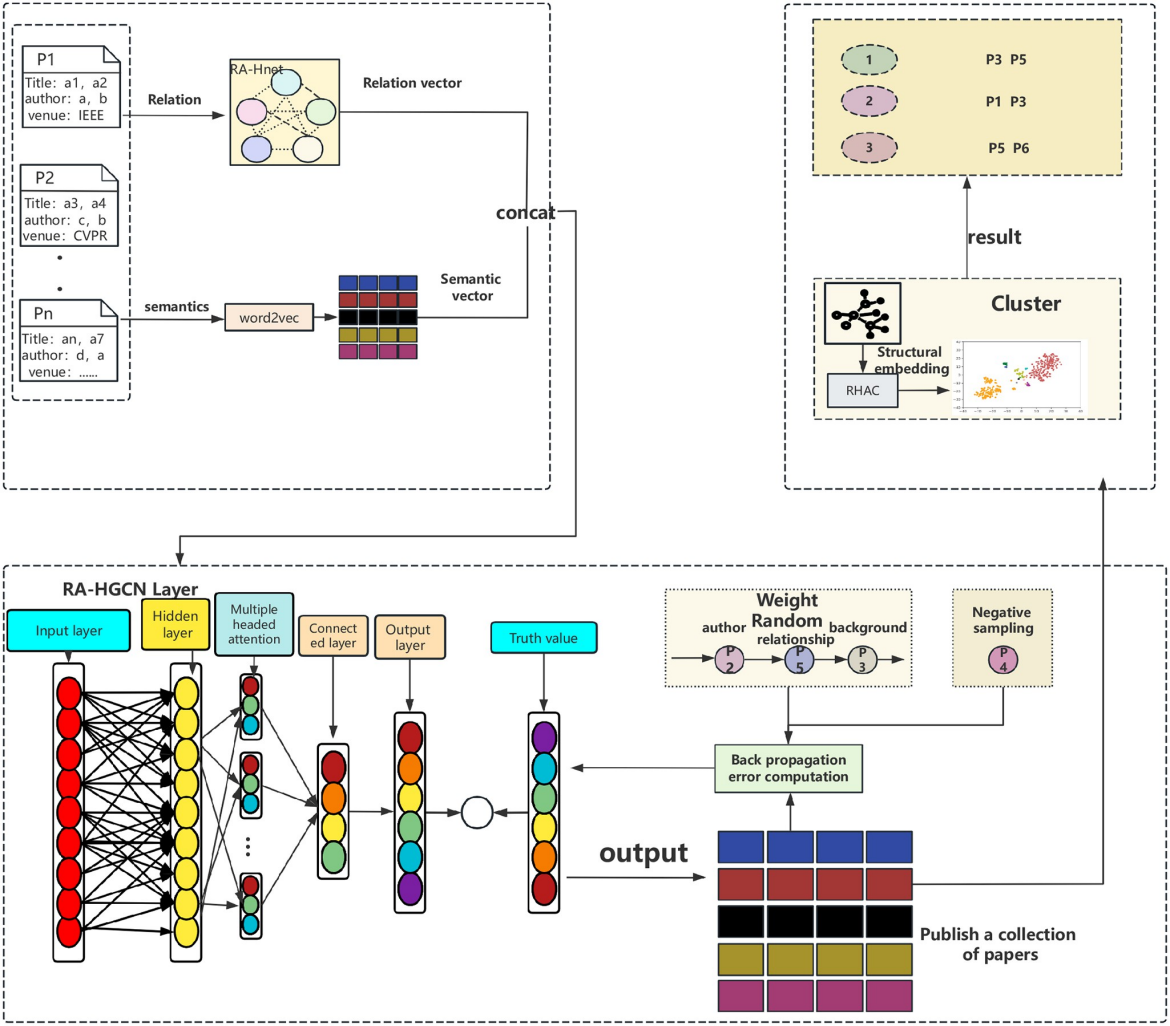
RA-HGCN network model.

Where *k* represents the size of the context association, $C_{i}^{k}$ is the set of *k* context nodes before and after in the path set. In a random traversal of the set, different sets of association sizes represent different neighbouring nodes. λ Parameters control overfitting. The general framework of the RA-HGCN network model in this paper is shown in Fig 4, and the model mainly differs from the classical network embedding methods in the following ways: (1) The model in this paper can preserve various heterogeneous relationships between nodes as well as relationship weights. (2) Using the weighted heterogeneous model in combination with the network, it is able to fuse the semantic and relational features of the published papers, and then embedded into the network afterwards, which is able to obtain better disambiguation effect.

### Graph-based hierarchical clustering

Through the investigation, this paper found that the published papers have a few authors published a large number of papers of the situation, and the hierarchical clustering (HAC) is applied in many references, but there are also the following shortcomings: HAC needs to pre-set the number of K clusters, and thus the algorithm’s time complexity is high, and how to determine the value of K is also a difficult problem, therefore, we improved the original hierarchical clustering (RHAC) Therefore, we improved the original hierarchical clustering (RHAC) by introducing the topology of the graph and the relationship between the graph nodes to reduce the computational complexity in hierarchical clustering and improve the efficiency.

For a graph G=(V,L,R), the weights of *l*_*ij*_ ∈ *L* of the edges between node and node *v*_*i*_ are denoted as, As shown in the following Eq (13):

$$\begin{array}{c} |l_{ij}|=\sigma \left(u_{i}\cdot u_{j}\right)\cdot \delta \left(\left(p_{i}\cdot p_{j}\right)\in L\right) \end{array}$$ (13)

Where *σ*(⋅) is the function with the range (0, 1) and *δ*(*a*) is a truth value function which is 1 when a is true and 0 otherwise.In RHAC, we first consider each sample as a separate cluster and then use the idea of hierarchical clustering to merge the two clusters which have the largest similarity at each step until they are merged to the specified K-value, and when the K-value is not determinable, we use the optimal module partitioning mechanism to determine it automatically partition of the paper, the node partitioning module is as follows, As shown in the following Eq (14):

$$\begin{array}{c} M=\frac{1}{2m}\sum_{p_{i},p_{i}\in V}\left(|l_{ij}|-\frac{w_{i}w_{j}}{2m}\right)\delta \left(b_{i}=b_{j}\right) \end{array}$$ (14)

Where *m* represents the sum of edge weights in the graph G, *w*_*i*_ represents the sum of weights of all edges of the node *p*_*i*_, and *b*_*i*_ represents the cluster *p*_*i*_ where is located; In brief, in our RHAC, after each merging process, the current module division degree *M*, is calculated until the final calculation is completed, and the largest *M* value is selected as the K value, after which the clustering result is calculated. In RHAC, the use of vector operations instead of the original distance calculation improves the efficiency and accuracy of clustering, and we have conducted sufficient comparative experiments in our experiments.

## Experimental results

In this section, this paper demonstrates the effectiveness of the newly proposed framework in the task of author name disambiguation, and we conduct experiments using the Aminer-v1, Aminer-v2, and WhoISWho datasets, and the results show that the framework approach in our work has a significant advantage over the other classical approaches.

### Dataset

**Fig 5:**
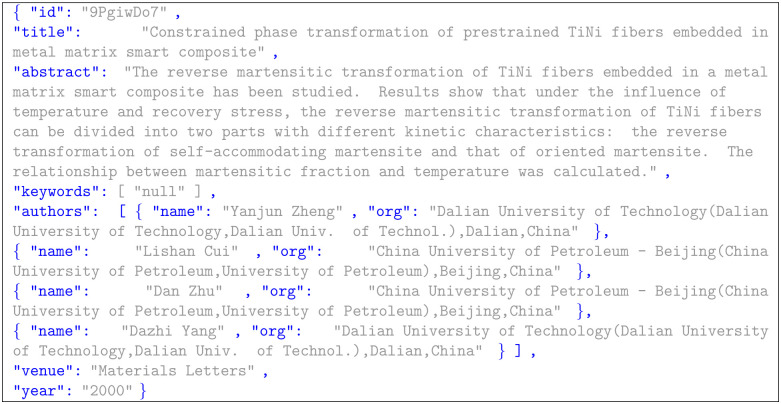
Examples of data sets.

In order to test the method proposed in this paper, we used the three Aminer [1] dataset datasets mentioned above. The Aminer dataset is widely used in the field of author name disambiguation, and the names of the people in the dataset are fully labeled with information about the titles, abstracts, authors, and institutions of the literature, including 8505 published papers, 1703 authors, 1977 conferences (journals) and 110 fuzzy names, etc. AMiner-v2 includes 70285 published papers, 12798 authors, and 100 fuzzy names. whoIsWho includes 399255 published papers, 45187 authors, and 421 fuzzy names, and the following Fig 5 shows an example of the data.

### Baseline methodology

In order to test the effectiveness of the RA-HGCN proposed in this paper for the task of author name disambiguation, the following classical methods are used in this paper to compare with our method. These methods are briefly explained:

RA-HGCN: Our approach considers the relations and semantics of published papers and constructs a heterogeneous graph convolutional neural network based on relations and semantics, and finally clusters using RHAC to obtain results.

Node2Vec [21]: This approach samples the sequence of neighbouring nodes of each node through a random wandering algorithm, and then processes the node sequence using a Word2Vec-like approach for the final network node representation.

Struct2Vec [22]: This approach considers the degree of structural similarity of two nodes in the relationship graph, even if these two nodes are far apart, if the structures are more similar, their similarity is correspondingly higher.

GHOST [23]: This approach constructs a collaborative network graph for each author name with ambiguity and calculates the similarity using a path-based approach, and then uses affinity propagation clustering to obtain the disambiguation results. The proposed affinity propagation algorithm is a clustering algorithm that does not require the number of clusters to be specified similar to the clustering in this paper.

Zhang et al [3]: This approach is based on automatic coding of graphs and embedding of publication paper nodes with local connections learnt globally with metrics, after which an end-to-end model is constructed and trained using neural networks, and finally hierarchical clustering is used to determine the allocation of publications.

Xu et al [24]: This method mainly deals with the part of authors with ambiguous names, builds multiple publishing networks for multiple relations respectively, after that uses a merging strategy for coarsening on each network separately, learns and embeds them by employing a newly proposed network embedding method, and finally determines the final clustering results using the (Density Clustering) and (Graph Based Clustering) algorithms.

Zhang et al [18]: In order to facilitate the processing of the relationships between the data, this method constructs three independent networks for each published paper with dichotomies, the processed relationships are learnt using network embedding, and finally hierarchical clustering is used to determine the final result.

DeepWalk [25]: This method is an approach to learn potential relationships between nodes by using a random walk strategy and use it for network embedding, this method is mainly applied in homogeneous unweighted networks.

LINE [26]: This method is widely used in isomorphic weighted networks and is able to keep the first-order and second-order information of the nodes similar.

SDNE [27]: This method can be viewed as an extension of the LINE method, but unlike the LINE algorithm, SDNE optimises both the first-order and second-order similarities, where the first-order similarity is the similarity between vertices directly adjacent to each other, and the second-order similarity is the similarity between the neighbouring nodes of the vertices, and the node embeddings obtained from the neighbouring nodes are close to each other in the space by optimising the two similarities at the same time.

Metapath2Vec [28]: This approach is widely used in heterogeneous networks in the presence of binary edges, where a neighbourhood is constructed for the current node by using random wandering of metapaths, followed by embedding using a heterogeneous jump model.

Hin2Vec [20]: This method is also an unweighted heterogeneous network embedding method that preserves the semantics of the relationships between nodes and the details of the network structure, and in this way enhances the learning between the nodes.

GraphSAGE [19]: This method performs node embedding by using different aggregation functions to form node neighbourhoods, but this method does not use unsupervised loss functions and thus can only be applied with isomorphic networks.

Qiao [8]: This approach is done by constructing a heterogeneous graph convolutional network and using a clustering algorithm for disambiguation, but this approach does not take into account the treatment of relationships between nodes.

### Experimental setup

In our experiments, we only used three features of the published paper as the presentation, and thus may have been less effective than the presentation of the original paper. in order to facilitate the comparison of experiments, We set the number of clusters of clustering set to a real number, and use the accuracy (Pairwise Precision), recall (Pairwise Recall) and *F*_1_ − *Score* [11] as the main evaluation metrics for the effectiveness of our experiments As well as the baseline methodology experiments, we also computed the *Macro*_1_ − *Score* (representing the average *F*_1_ value of all ambiguous names) metric for the dataset for comparison, As shown in the following Eqs (15), (16) and (17).

$$\begin{array}{c} \text{precision}=\frac{\text{TP}}{\text{TP}+\text{FP}} \end{array}$$ (15)

$$\begin{array}{c} \text{Re}call=\frac{TP}{TP+FN} \end{array}$$ (16)

$$\begin{array}{c} F_{1}=\frac{2\times Precision\times Recall}{Precision+Recall} \end{array}$$ (17)

Where *TP* denotes the number of papers predicted to be by the same author and actually by the same author, *FP* denotes the number of papers predicted to be by the same author but actually not by the same author. *FN* denotes the number of papers predicted to be by different authors but actually by the same author. In calculating the value of *F*_1_, we choose the harmonic mean of precision and recall, and the value ranges between (0, 1), with higher values indicating a more effective model.

We use one RTX A5000 GPU to train the model and use the pre-trained wod2vec model to handle the embedding of thesis node relationships, in terms of parameters we use one RTX A5000 GPU to train the model and use the pre-trained wod2vec model to handle the embedding of thesis node relationships, in terms of parameters we set the initial learning rate to, the regularization factor, linked between above, because of the difference in dataset sizes, by experimenting with the embedding dimensions we chose 128 embedding dimensions on the V1 dataset, and 256 dimensions as well as 512 dimensions on V2 as well as WHOISWHO datasets, respectively., we set the initial learning rate to, the regularization coefficient, the link between the above because of the difference in dataset sizes, by experimenting with the embedding dimensions we choose an embedding dimension of 128 on the V1 dataset, and 256 dimensions as well as 512 dimensions on the V2 as well as WHOISWHO datasets, respectively.

### Results

Table 1 shows the average of the results for each metric, which is obtained by averaging all the different disambiguation names by summing them up, and it can be seen from the table that the method in this paper is basically superior to the other methods, e.g., it outperforms the other baseline methods on the Avg Pre metrics by 1.42 to 31.28 (SDNE+31.28, Struct2Vec+6.95, Aminer+5.08, Node2Vec+12.38, Zhang et al+12.41, GHOST+1.42, and GHOST+1.42). 5.08, Node2Vec+12.38, Zhang et al+12.41, GHOST+1.42). In addition to the metrics comparison of several classical methods, we also performed experimental comparisons of several other baseline methods mentioned, the results of which are shown in Table 2 below. Our method achieved the best results in both Pre metrics and F1 metrics, Line uses a homogeneous network that takes into account first-order and second-order relationships, PHNet uses a heterogeneous network for inter-local learning, BOND’s method works by combining semantic features with heterogeneous graphs, and our method takes into account the relationship between semantics and relations. And the combination of the two greatly improves the expressiveness of the features, which makes our method achieve better results in all three types of metrics. In the metrics of Rec, our method also achieved the second best result, while the first one, Xu’s method, was able to achieve better recall more because it mainly focuses on the gap between positive and negative edges and learns the global graph attributes in a coarsened network, about our method achieved a high recall as well as F1 score, but the accuracy is relatively low, we analyze that it may be due to the fact that We capture the complex and diverse relationships between authors by using a heterogeneous graph convolutional attention network, which contributes to the recall rate. Meanwhile, as the multi-head attention mechanism can improve the model’s ability to learn to understand complex graph structures, which helps the F1 score. And regarding the relatively low accuracy rate may be due to the fact that there is a category imbalance among the unused authors, i.e., some authors have more samples while others have fewer samples.

**Table 1:** Comparison of the methodology of this paper with several classical methods for the three types of indicators.

Method	Our Method	SDNE	Struct2Vec	Aminer	Node2Vec	Zhang et al	GHOST
Pre	**83.04**	51.76	76.09	77.96	70.66	70.63	81.62
Rec	81.43	**90.74**	30.33	63.03	65.39	59.53	40.43
F1	**81.34**	59.95	39.76	69.70	61.93	64.61	54.07

**Table 2:** Comparison of this paper’s method with ten baseline methods on the Aminer dataset for three types of metrics.

Name	Our Method	PHNet	BOND	Xu	Hin2Vec	Component	DeepWalk	LINE	Metapth2Vec	GraphSAGE
Pre	**83.04**	65.91	70.21	74.5	61.6	58.2	77.8	73.7	63.0	79.03
Rec	81.43	68.32	72.78	**83.2**	74.3	73.4	69.5	59.7	60.3	70.19
F1	**81.34**	67.09	71.4	63.5	56.2	48.2	73.4	66.1	61.6	74.34

In order to more visually demonstrate the superiority of the methodology of this paper and the effectiveness of the comparative methodology on specific names, for individual names, these names are often listed as classic examples for citation illustration in previous studies. we give the name disambiguation results of our model and several other baseline models for some specific authors on the Aminer dataset in Table 3 below. In the Table 3, we have selected ten of the names and computed the average scores; for example, Bin Yu belongs to six different authors in the dataset, and five of them have published only one paper; similarly, the names of each of them in the figure represent multiple different authors, and a portion of the authors (about 46%) have published only one paper in their entire study career, and similarly, there are there is a proportion of authors who have published a large number of papers during their study career. The table below shows the *F*_1_ scores for the names shown, with the last row (Avg) representing the scores for all the names on the macro level, being the average of the scores for all the different names.

**Table 3:** Comparison of the effectiveness of this paper’s method with ten baseline methods for disambiguation on the Aminer dataset.

Name	Our Method	Qiao	Zhang	Zhang	Xu	Hin2Vec	Component	DeepWalk	LINE	Metapth2Vec	GraphSAGE
Ajay Gupta	0.697	**0.750**	0.618	0.568	0.552	0.684	0.329	0.370	0.578	0.298	0.654
Alok Gupta	**1.000**	0.942	0.590	0.689	0.892	0.734	0.690	0.582	0.835	0.663	0.651
Bin Yu	**0.987**	0.696	0.614	0.431	0.585	0.490	0.292	0.490	0.475	0.354	0.441
David Cooper	0.900	0.900	**0.931**	0.737	0.884	0.931	0.327	0.737	0.833	0.833	0.862
David Nelson	**0.944**	**0.944**	0.556	0.750	0.735	0.635	0.219	0.353	0.523	0.788	0.710
Fei Su	**1.000**	**1.000**	0.941	0.933	0.630	0.917	0.648	0.684	0.721	0.930	0.948
Hao Wang	0.687	0.604	0.543	0.403	0.557	**0.624**	0.086	0.382	0.400	0.420	0.192
Jie Tang	**0.989**	0.982	0.910	0.657	0.522	0.825	0.883	0.738	0.432	0.902	0.741
Thomas Wolf	0.720	**0.860**	0.352	0.703	0.522	0.516	0.502	0.320	0.357	0.390	0.710
Yang Wang	**0.989**	0.548	0.409	0.273	0.574	0.443	0.118	0.171	0.211	0.310	0.204
Avg	**0.813**	0.786	0.680	0.715	0.681	0.629	0.507	0.563	0.606	0.643	0.678

As shown by the experimental results in the table above, the method proposed in this paper is significantly better than all the baseline methods, e.g., the average score (Avg) is higher than the other baseline methods by 0.048 to 0.327 (Component+0.048, Zhang+0.119, DeepWalk+0.271, LINE+0.228, Qiao+0.048, etc.).

In individual names, other baseline methods also slightly outperform this paper’s method, e.g., in the results for the name David Cooper, Zhang et al.’s method achieves a score of 0.931, which may be attributed to the fact that the baseline method’s end-to-end local joins are better at disambiguating the name David Cooper, whereas in the results for the name Thomas Wolf, the Qiao et al. achieved a score of 0.860 better than the method of this paper 0.72.

It has been proved experimentally that the method proposed in this paper is significantly better than all the baseline methods, which may be due to the fact that, in this paper, the use of a combination of semantics and relations for embedding is able to improve the learning ability of the network. In terms of the network, the model in this paper has a significant advantage over the DeepWalk model in terms of embedding due to the use of random sampling of association weights of meta-paths as well as a semantic and relationship-based jump model; similarly, the model in this paper takes into account the relationships and node types in the network as well as the different weights of the relationships, which makes it stronger than the LINE, in terms of learning. Metapth2Vec model.

In order to evaluate the effectiveness of this paper’s method on different datasets, this paper also uses three datasets for comparison experiments, and the results of the experiments are shown in Table 4. From Table 5, we can see that the results of this paper’s method on different datasets are also basically superior to the baseline method. GHOST constructs a partnership network for each author with ambiguities, which may have made the prediction accuracy of their method higher, whereas this paper’s method basically takes all three metrics into account, and collectively, this paper’s method outperforms the other baseline methods.

**Table 4:** Comparison of ablation experiments.

Method	HAC	K unknown	K known
Pre	85.95	51.01	83.04
Rec	76.46	95.46	81.43
F1	79.48	58.78	81.34

**Table 5:** Comparison of the methods in this paper on three different datasets.

Method	AMiner-v1			AMiner-v2			WhoISWho		
	P	R	F1	P	R	F1	P	R	F1
AMiner	82.36	79.23	80.76	77.56	63.44	69.79	56.29	48.03	51.83
GHOST	**93.46**	67.49	78.38	**85.13**	43.28	57.39	**76.16**	24.59	37.18
DeepWalk	80.54	74.67	77.49	57.92	62.31	60.03	24.37	**68.18**	35.91
Node2Vec	64.51	**73.48**	68.70	28.45	**57.96**	38.17	31.64	62.21	41.95
Our method	83.04	81.43	**81.34**	75.64	67.90	**71.56**	58.19	69.89	**63.47**

### Experimental setup

**Fig 6:**
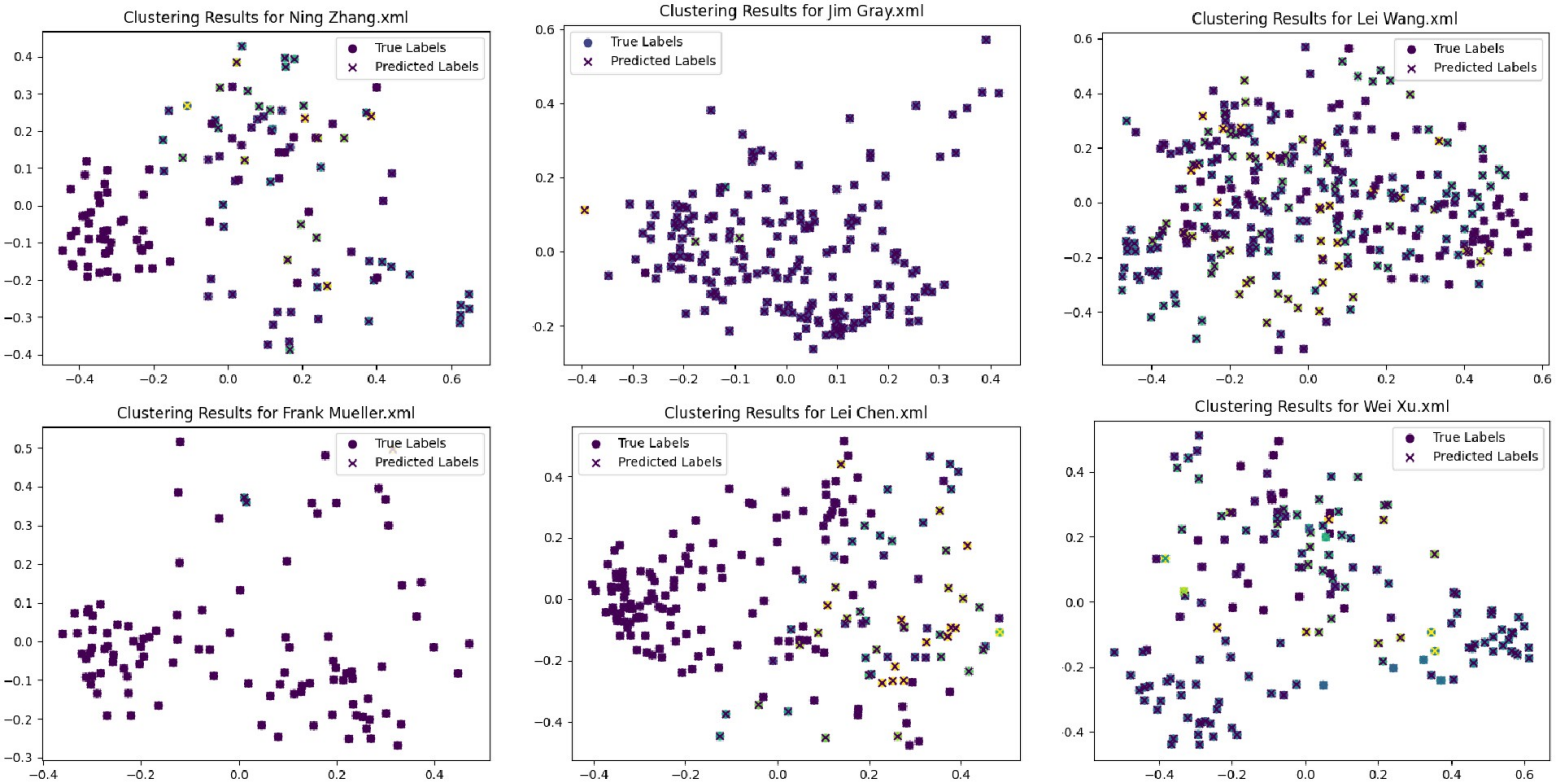
Comparison of actual and predicted clustering of RHAC in six different names. The origin of the figure shows the actual clustering effect, and the × sign shows the predicted clustering effect.

In this paper, we have used Improved Hierarchical Clustering (RHAC) and shown the predicted classification of some of the names in the Aminer dataset in Fig 6. As can be seen from the figure below, most of the nodes are accurately predicted in the clustering results of RHAC, which proves that the method of this paper is more superior when compared to the classical clustering methods.

**Fig 7:**
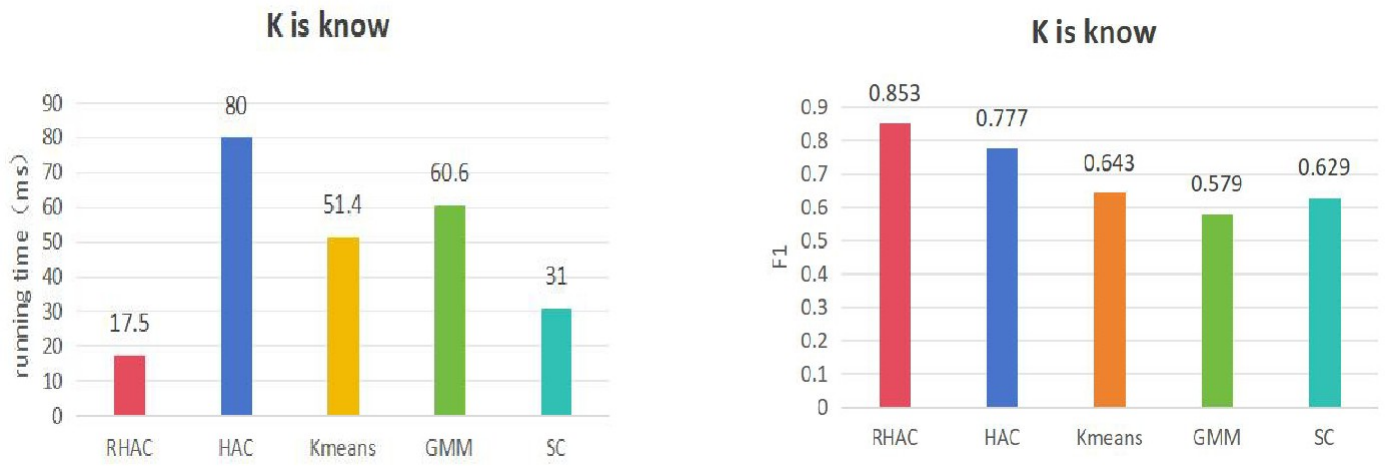
Comparison of time consumed by different clustering algorithms when K value is known and the score.

**Fig 8:**
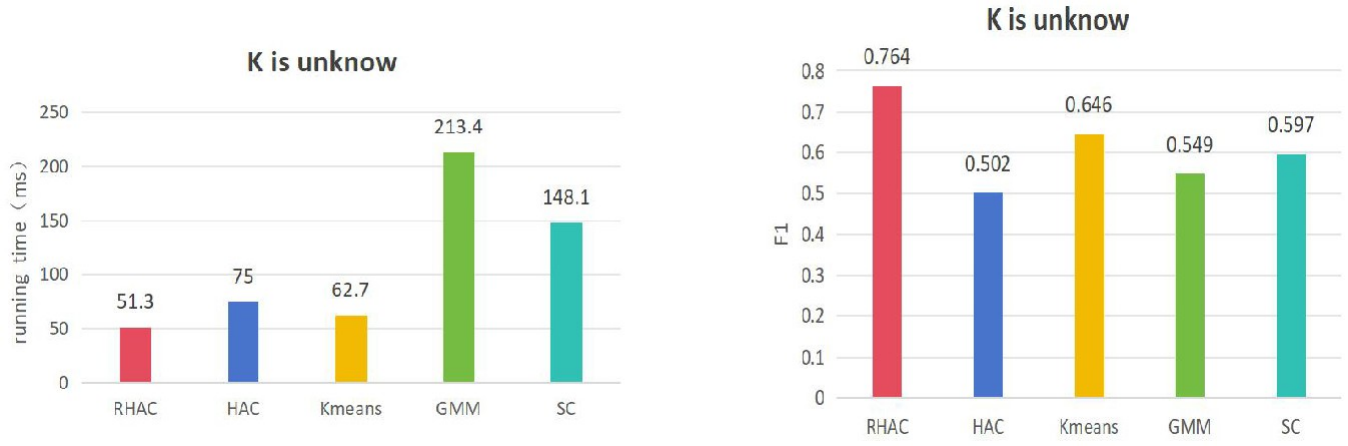
Comparison of time consumed by different clustering algorithms when K value is known and the score.

In order to further validate the superiority of the clustering algorithm (RHAC) in this paper, this paper also compares the time consumed by clustering with four classical clustering algorithms, namely hierarchical agglomerative clustering (HAC) K-means, Gaussian Mixture (GMM), and Spectral Clustering (SC), respectively, in terms of the K-value is known, and the K-value is unknown. The results of the comparison of the K-value is known in the following Fig 7. From the experimental results in Figs 7 and 8, it can be seen that the clustering efficiency as well as the clustering results of this paper’s clustering algorithm, RHAC, are better than other classical clustering algorithms on the Aminer dataset, which suggests that this paper’s introduction of the topology of the graph and the relationships between nodes into hierarchical clustering to improve the efficiency of traditional clustering is effective.

**Fig 9:**
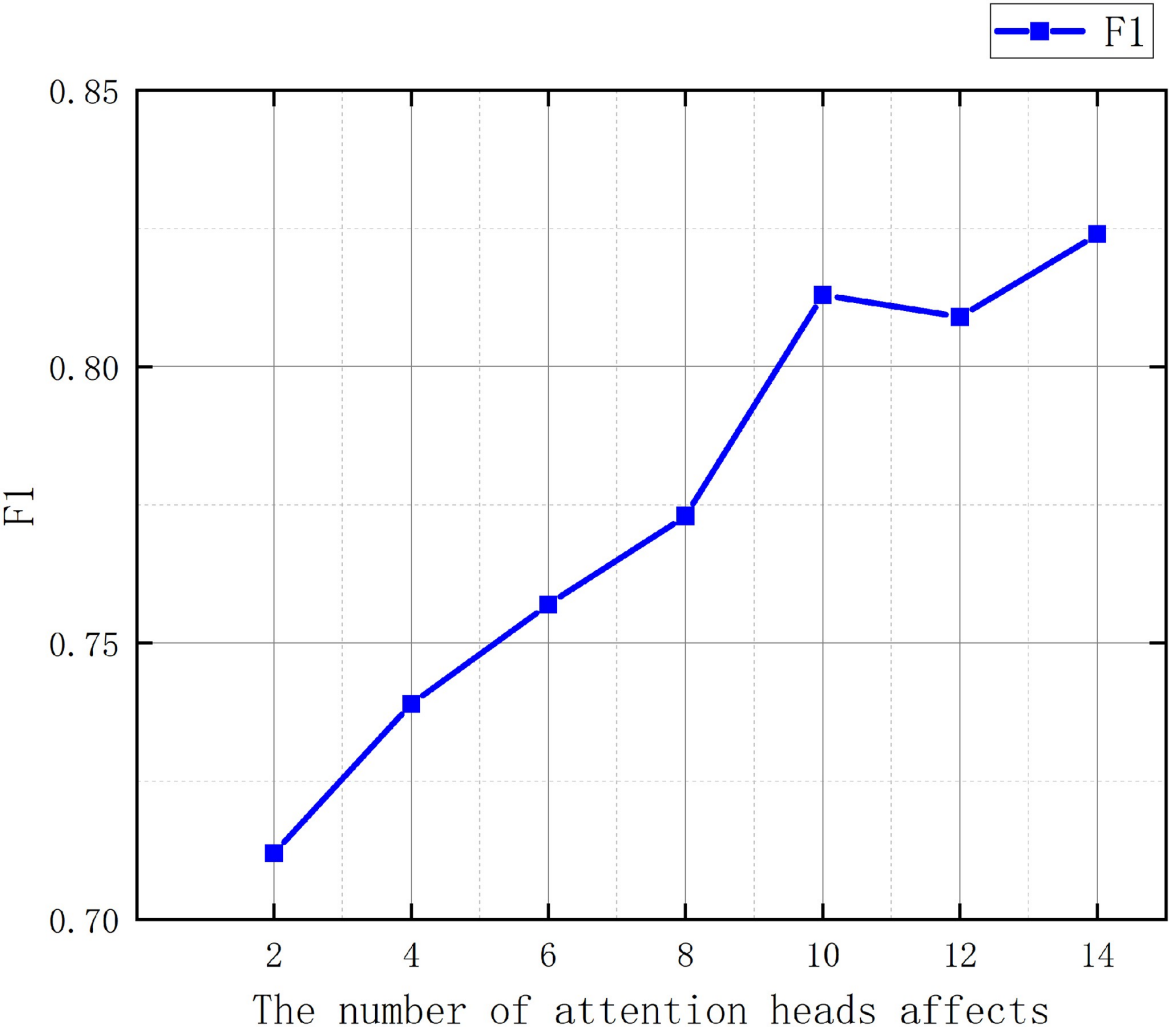
The effects of the number of attentional heads on the experimental results.

**Fig 10:**
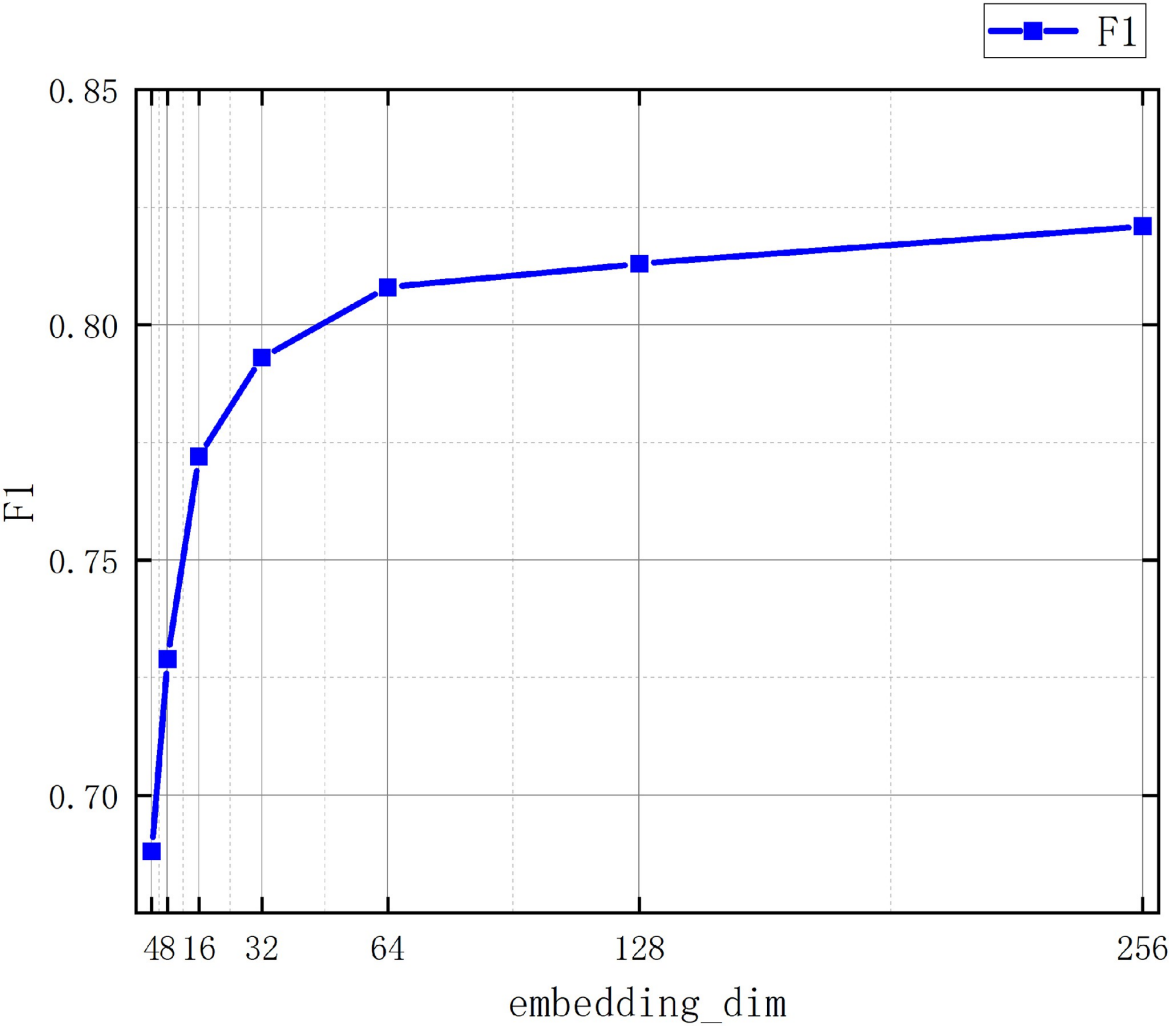
The effects of the number of attentional heads on the experimental results.

Ablation experiments:

In this paper, ablation experiments were conducted on the improved graph clustering algorithm by comparing it without specifying k-value using graph convolution module, specifying k-value using graph module and with classical hierarchical clustering, and the results are shown in Table 4 below: As can be seen from the experimental results in Table 4, the improvement of classical hierarchical clustering using the graph convolution module in this paper achieves better results in a comprehensive way, the recall of this paper’s method reaches 95.46, higher than that of the classical method by 19.00 when the value of k is not specified, and when the value of k is specified, the value of f1 of this paper’s method reaches 81.34, higher than that of the classical method by 1.86, and the accuracy and recall scores are both higher than 80, which proves that the improvement strategy of this paper is effective. In this paper, we also conducted ablation experiments on the newly added multi-head attention mechanism. The settings of the number of attention heads are (2, 4, 6, 8, 10, 12, 14), the results are shown in Fig 9, through the experiment on the number of attention heads, it is concluded that the F1 value gradually improves with the increase of the number of attention heads and gradually stabilizes when the number of attention heads reaches 10, and the subsequent fluctuation of the F1 value with the increase of the number of attention heads may be due to the fact that, with the increase of the number of attention heads, the too many attention heads cannot have a significant effect on the improvement of the experimental effect, and also greatly improves the structural complexity of the network and improves the time of training, therefore, in this paper, we choose to use 10 attention heads as the embedding of multi-head attention. In this paper, we also conducted experiments on the embedding dimension of the parameters, the embedding dimension is set as (4, 8, 16, 32, 64, 128, 256, 512) The experimental results are shown in Fig 10, and the F1 value is gradually improved with the increase of the embedding dimension; in the V1 dataset, the model reaches a relative stability in the 128-dimension, and there is a slight decrease on the experimental results after the 128-dimension, which may be due to the embedding of the dimension is too large that leads to too much information embedded, which has an effect on the experimental results. In the V2 dataset because the amount of data is much larger than V1, the V2 dataset reaches relative stability at 256 dimensions. In the WHOISWHO dataset, the model reaches relative stability at 512 dimensions. Therefore, this paper uses 128 dimensions for the V1 dataset, 256 dimensions for the V2 dataset, and the same 256 dimensions for the WHOISWHO dataset as the dimension embedding.

## Conclusion

In this paper, we propose a new Heterogeneous Graph Convolutional Attention Network (RA-HGCN) embedding and using Improved Hierarchical Clustering Disambiguation (RHAC) for author name disambiguation. Our network not only solves the problem that isomorphic graphs do not fully utilize the node information of the graph, but also considers the co-authorship problem. We give a threshold value between the authors of an article, and the authors who co-authored a paper that exceeds the threshold value are defined as reliable co-authors, and use the author name disambiguation method based on the relational graph heterogeneous neural network, firstly, the semantic and relational information of the published papers will be extracted and the information will be trained to be spliced using our graph convolution module, and a better representation of the features can be obtained by processing the information, and then it is inputted into our constructed relational heterogeneous graph attention neural network for training to get the vector representation of semantic and relational information. Finally, we use the improved hierarchical clustering algorithm (RHAC) to combine the relationship and topology between the graphs such as relational semantics and hierarchical clustering, and use the vector representations obtained from the training to replace the distance calculation of the original hierarchical clustering, so that our clustering method can determine the optimal k-value automatically during the clustering process, and improve the accuracy and efficiency of the clustering. After experiments, the average F1 value of this paper’s method on the Aminer dataset using only three thesis features is 0.834, which is higher than other mainstream methods. Comparisons with several baseline methods and ablation experiments on the proposed method show that the method proposed in this paper significantly outperforms the baseline method in terms of accuracy as well as consumption time. However, the method proposed in this paper is not considered separately for newly published papers i.e., for incremental ablation, which also makes it necessary to reconstruct the network for training for newly published papers, which greatly increases the time required for ablation. In our future work, we will try to improve our method so that it can effectively disambiguate the increments.
